# Supplementary material for: Risk factors associated with the development of delirium in general ICU patients. A prospective observational study
Source: PLoS One. 2021 Sep 2;16(9):e0255522. doi: 10.1371/journal.pone.0255522 (PMC8412262; doi:10.1371/journal.pone.0255522)
Supplement: S1 Table — (DOCX) [file pone.0255522.s001.docx]

**S1 Table. Univariate analysis**

| **Variable** | **OR (95% IC)** | **P value** |
| --- | --- | --- |
| Age > 74 yrs | 1,92 (1,24-2,98) | 0,003 |
| Clinical Frailty Scale > 3 | 1,80 (1,36-2,39) | 0,0002 |
| Emergency surgery or medical patient | 1,95 (1,15-3,31) | 0,001 |
| Acute respiratory failure as main diagnosis on admission | 2,56 (1,54-4,25) | 0,0002 |
| Sepsis as main diagnosis on admission | 1,86 (1,21-2,86) | 0,006 |
| Coma as main diagnosis on admission | 3,38 (1,59-7,19) | 0,0008 |
| Cardiac arrest as main diagnosis on admission | 3,19 (1,06-9,59) | 0,03 |
| Cardiovascular comorbidities | 1,56 (1,01 a 2,42) | 0,04 |
| Renal comorbidities | 3,60 (2,35-5,51) | <0,0001 |
| Endocrine comorbidities | 1,23 (0,99-1,53) | 0,09 |
| SAPS-3 score > 56 | 4,07 (2,65-6,23) | <0,0001 |
| SOFA score on admission > 4 | 4,05 (2,64-6,21) | <0,0001 |
| Invasive mechanical ventilation | 4,81 (3,10 a 7,44) | <0,001 |
| Reintubation | 5,93 (2,07 a 17,00) | <0,001 |
| Preventive isolation on suspicion of MDR | 2,00 (1,42 a 2,81) | <0,001 |
| Confirmed isolation on suspicion of MDR | 4,01 (2,14 a 7,49) | <0,001 |
| Need of prone position | 7,64 (2,26-25.88) | <0,001 |
| Neuromuscular blockade | 18,81 (5,63-62,87) | <0,001 |
| Organ failure during ICU admission: cardiovascular | 6,78 (4,18-11,01) | <0,001 |
| Organ failure during ICU admission: respiratory | 3,95 (2,52-6,19) | <0,001 |
| Organ failure during ICU admission: renal | 3,60 (2,35-5,52) | <0,001 |
| Organ failure during ICU admission: hepatic | 3,11 (1,65-5,87) | <0,001 |
| Organ failure during ICU admission: hematologic | 1,88 (1,10-3,21) | 0,02 |
| Number of organ failures > 2 | 8,55 (5,48-13,44) | <0,001 |

Yrs = years; ICU = intensive care unit; MDR = multidrug-resistant bacteria
