# Supplementary material for: Risk factors associated with the development of delirium in general ICU patients. A prospective observational study
Source: PLoS One. 2021 Sep 2;16(9):e0255522. doi: 10.1371/journal.pone.0255522 (PMC8412262; doi:10.1371/journal.pone.0255522)
Supplement: S1 File — (DOCX) [file pone.0255522.s002.docx]

**Abbreviations**

**ABCDEF bundle** = Assess, Prevent, and Manage Pain, Both Spontaneous Awakening Trials (SAT) and Spontaneous Breathing Trials (SBT), Choice of analgesia and sedation, Delirium: Assess, Prevent, and Manage, Early mobility and Exercise, and Family engagement and empowerment.

**APACHE** = Acute Physiology and Chronic Health Evaluation

**CAM-ICU** = Confusion Assessment Method for the Intensive Care Unit

**CFS** = Clinical Frailty Scale

**CHAID** = Chi-square automatic interaction detection

**MDR** = multi-drug resistant pathogen

**MV** = mechanical ventilation

**NMB** = neuromuscular blockade

**OF** = organ failure

**OR** = Odds Ratio

**PADIS** = Pain, Agitation/Sedation, Delirium, Immobility, and Sleep

**SAPS-3** = Simplified Acute Physiology Score - 3

**SOFA** = Sequential Organ Failure Assessment

**RASS** = Richmond Agitation Sedation Scale

**ICU** = intensive care unit
