# Supplementary material for: Risk factors associated with the development of delirium in general ICU patients. A prospective observational study
Source: PLoS One. 2021 Sep 2;16(9):e0255522. doi: 10.1371/journal.pone.0255522 (PMC8412262; doi:10.1371/journal.pone.0255522)
Supplement: Attachment — Submitted filename: R1_PONE-D-21-09224.docx [file pone.0255522.s003.docx]

Risk factors associated with the development of delirium. A prospective observational study.

Dear Dr. Zivkovic,

In this manuscript, the authors investigated risk factors associated with the development of delirium in an ICU cohort. As results they described an incidence of 6.3% in their population. Patients with delirium were older, had higher Clinical Frailty Scale, higher risk scores on admission and more organ failures compared to patients without delirium. Delirium in ICU patients is a relevant topic of high interest with the specialty that preventive measures are very helpful. I thank the authors for their contribution in this important field of research. Nevertheless, there are some aspects I would like to address.

Major and minor concerns:

- Current Guidelines describe a meta-analysis of existing risk factors from a great number of studies and cohorts. So, the topic isn’t new and a lot of studies on delirium risk factors are available. The reported risk factors in this study aren’t innovative and go in line with current guidelines. A systematic comparison with risk factors from current guideline could be an additional result to add value to the manuscript.
- A delirium incidence of 6.3% on an ICU is comparably low. Regarding a sensitive delirium screening (3 times a day with CAM-ICU) the authors should describe their population more precisely, also the group of patients without delirium. Maybe their ICU population has lower risk for delirium compared to other populations (e.g. no heart surgery patients).
- Who performed Delirium screening and Clinical Frailty Scale? Are these routine data from clinical staff?
- Please put data from univariate analysis at least in a supplement.
- The discussion should focus more on the results from multivariate analysis. The conclusion that sicker and older patients have a higher risk for delirium is too little, because it is know from preexisting literature. What is new? What is the difference? What implementation strategies will be made to prevent delirium on their ICU?

It is a simple, well-executed prospective cohort description. Perhaps the authors can put some more work into comparing them to the existing literature and highlighting the specifics of their work.

Best regards

Anika Müller
